# Clinical Characteristics of Combat Crewmen

**DOI:** 10.1001/jamanetworkopen.2026.2032

**Published:** 2026-03-16

**Authors:** Jacob R. Powell, Sara M. Lippa, Tamara L. McKenzie-Hartman, Chandler Sours Rhodes, Treven C. Pickett, Rujirutana Srikanchana

**Affiliations:** 1 The National Intrepid Center of Excellence, Walter Reed National Military Medical Center, Bethesda, Maryland; 2 General Dynamics Information Technology, Falls Church, Virginia; 3 Neuroscience Program, Uniformed Services University of the Health Sciences, Bethesda, Maryland; 4 Department of Diagnostic Radiology and Nuclear Medicine, University of Maryland School of Medicine, Baltimore; 5 Department of Medical and Clinical Psychology, Uniformed Services University of the Health Sciences, Bethesda, Maryland; 6 Department of Physical Medicine and Rehabilitation, Uniformed Services University of the Health Sciences, School of Medicine, Bethesda, Maryland

## Abstract

This cross-sectional study examines clinical characteristics of traumatic brain injury and the psychological health of combat crewman and special operators to determine how brain health risks can be detected and mitigated.

## Introduction

As part of maritime special operations, combat crewmen perform high-risk boat operations and training over extended time periods. They operate vessels traveling up to 40 knots, exposing them to repetitive wave impacts and vibration forces over 20G.^1^ Media attention has raised concerns about brain health risks associated with high-speed vessel operations.^2^ Exposure consequences have drawn international attention, prompting an international task force to study health impacts and injury prevention strategies.^3^ Currently, most literature describing clinical characteristics of combat crewmen is limited to musculoskeletal injury characterization. There are scientific gaps regarding neurological and psychological health.^2^ This is concerning because one-third of a retired combat crewmen sample reported at least 1 instance of losing consciousness onboard and 70% reported impaired job performance capabilities due to forceful impacts.^4^ There is a need to understand how exposures sustained by combat crewmen influence clinical symptoms so brain health risks can be detected and mitigated. This study compares self-reported clinical symptoms between combat crewmen and special operators.

## Methods

This cross-sectional study followed the STROBE reporting guideline and used data collected from combat crewmen and special operators attending the National Intrepid Center of Excellence Intensive Outpatient Program for persistent clinical symptoms associated with traumatic brain injury (TBI) and psychological health conditions between 2011 and 2024. Participants provided informed consent. This study was approved by the Walter Reed National Military Medical Center Institutional Review Board.

We compared combat crewmen and special operators across demographics and self-reported symptom assessments collected from electronic records including postconcussive symptoms, posttraumatic stress, sleep, depression, anxiety, headache, and alcohol use reported at admission. Two-sided *t* tests compared differences in symptoms between groups using the false discovery rate to adjust for multiple comparisons. Cohen *d* effect sizes are reported. α level was set at *P* < .05. Data were analyzed from February to May 2025 using RStudio version 2023.3.0.386 (R Project for Statistical Computing).

## Results

Of 545 individuals (mean [SD] age, 39.80 [5.93] years), combat crewmen (86 [15.8%]) and special operators (459 [84.2%]) did not significantly differ in mean age or time in service (Table 1). Compared with special operators, combat crewmen reported significantly greater mean (SD) symptom severity on the NSI somatic sensory (8.73 [4.39] vs 7.49 [4.25]; *P* = .03), cognitive (9.15 [3.32] vs 8.05 [3.63]; *P* = .03), and vestibular (3.06 [1.92] vs 2.36 [1.99]; *P* = .01) subscales. No participants failed the Validity-10 scale within the NSI (identifying symptom overreporting based on endorsement of items unusual or unlikely following mild TBI).^5^ Total mean (SD) PCL-M scores were lower in combat crewmen (39.52 [13.28] vs 43.82 [14.37]; *P* = .04). The mean (SD) PCL-M reexperiencing (8.61 [3.94] vs 10.58 [4.74]; *P* = .01) and avoidance (14.81 [6.15] vs 17.26 [6.68]; *P* = .03) clusters differed significantly (Table 2). Combat crewmen reported a higher mean (SD) ESS (11.24 [5.06] vs 9.91 [4.94]; *P* = .04) and HIT-6 (55.61 [9.12] vs 52.89 [9.37]; *P* = .04), but lower AUDIT-C (3.16 [1.51] vs 3.83 [1.95]; *P* = .04).

**Table 1.** Demographics Characteristics by Occupational Specialty Group

Demographics	Patient, No. (%)^a^			*P* value	Corrected *P* value^b^
	All (N = 545)	Special operator (n = 459)	Combat crewman (n = 86)		
Age, mean (SD), y	39.80 (5.93)	39.79 (5.93)	39.86 (5.92)	.93	.93
Marital status					
Married	442 (81.1)	372 (81.0)	70 (81.4)	.40	.50
Single	52 (9.5)	47 (10.2)	5 (5.8)		
Divorced	30 (5.5)	23 (5.0)	7 (8.1)		
Separated	21 (3.9)	17 (3.7)	4 (4.7)		
Rank					
Enlisted	413 (75.8)	337 (73.4)	76 (88.4)	<.001^c^	.02^c^
Officer	132 (24.2)	122 (26.6)	10 (11.6)		
Race					
American Indian or Alaska	9 (1.7)	8 (1.7)	1 (1.2)	.02^c^	.05
Asian or Pacific Islander	9 (1.7)	7 (1.5)	2 (2.3)		
Other	12 (2.2)	6 (1.3)	6 (7.0)		
Unknown	33 (6.1)	28 (6.1)	5 (5.8)		
White	482 (88.4)	410 (89.3)	72 (83.7)		
Time in service, mean (SD), y	19.22 (5.63)	19.11 (5.56)	19.83 (5.94)	.19	.31

^a^ Percentages may not add up to 100% due to rounding.

^b^ The Benjamini-Hochberg false discovery rate corrected *P* value for occupational specialty group differences between combat crewmen and special operators for each corresponding demographic characteristic are reported in this column.

^c^ Statistical significance was set at *P* < .05.

**Table 2.** Clinical Self-Reports Scores by Occupational Specialty Group^a^

Self-report admission	All (N = 545)	Special operator (n = 459)	Combat crewman (n = 86)	*P* value	Corrected *P* value^b^	Cohen *d*^c^
NSI						
Total No.	517	436	81	NA	NA	NA
Total score	32.47 (13.06)	31.98 (13.07)	35.12 (12.74)	.04^d^	.06	0.24
Vestibular	2.47 (1.99)	2.36 (1.99)	3.06 (1.92)	<.001^d^	.01^d^	0.36
Somatic sensory	7.68 (4.29)	7.49 (4.25)	8.73 (4.39)	.01^d^	.03^d^	0.29
Affective	11.49 (4.97)	11.5 (5.02)	11.41 (4.71)	.84	.90	−0.02
Cognitive	8.22 (3.60)	8.05 (3.63)	9.15 (3.32)	.01^d^	.03^d^	0.31
PCL-M						
Total No.	393	331	62	NA	NA	NA
Total score	43.15 (14.27)	43.82 (14.37)	39.52 (13.28)	.02^d^	.04^d^	−0.30
Reexperience cluster	10.27 (4.67)	10.58 (4.74)	8.61 (3.94)	<.001^d^	.01^d^	−0.42
Avoidance cluster	16.87 (6.65)	17.26 (6.68)	14.81 (6.15)	.01^d^	.03^d^	−0.37
Arousal cluster	16.01 (4.83)	15.99 (4.78)	16.1 (5.10)	.98	.98	0.02
PSQI						
Total No.	513	430	83	NA	NA	NA
Total score	11.66 (3.76)	11.56 (3.78)	12.16 (3.67)	.17	.21	0.16
PHQ-9						
Total No.	508	425	83	NA	NA	NA
Total score	10.27 (5.44)	10.13 (5.49)	11.0 (5.17)	.15	.20	0.16
GAD-7						
Total No.	425	346	79	NA	NA	NA
Total score	10.23 (5.29)	10.17 (5.31)	10.47 (5.23)	.67	.78	0.06
ESS						
Total No.	526	442	84	NA	NA	NA
Total score	10.12 (4.98)	9.91 (4.94)	11.24 (5.06)	.03^d^	.04^d^	0.27
AUDIT-C						
Total No.	436	367	69	NA	NA	NA
Total score	3.72 (1.90)	3.83 (1.95)	3.16 (1.51)	.02^d^	.04^d^	−0.35
HIT-6						
Total No.	532	448	84	NA	NA	NA
Total score	53.32 (9.37)	52.89 (9.37)	55.61 (9.12)	.02^d^	.04^d^	0.29

Abbreviations: AUDIT-C, Alcohol Use Disorders Identification Test-Consumption; ESS, Epworth Sleepiness scale; GAD-7, General Anxiety Disorder-7; HIT-6, Headache Impact Test; NA, not available; NSI, neurobehavioral symptom inventory; PCL-M, PTSD Checklist Military Version; PHQ-9, Patient Health Questionnaire-9; PSQI, Pittsburg Sleep Quality Index.

^a^ Ranges of possible scores, cutoffs, and clinically relevant change (if known) may be found in the eTable in Supplement 1.

^b^ This column reports the Benjamini-Hochberg False Discovery Rate corrected *P* value for occupational specialty group differences between combat crewmen and special operators for each corresponding clinical self-reported outcome measures.

^c^ The Cohen’s *d* effect size is reported for each group comparison in this column indicting the standardized mean difference between combat crewmen and special operators for each self-reported outcome measure.

^d^ Statistical significance set at *P* < .05.

## Discussion

Combat crewmen reported increased somatic, cognitive, vestibular, sleepiness, and headache interference symptoms but decreased posttraumatic stress symptoms and alcohol consumption compared with special operators. Posttraumatic stress has been consistently associated with increased neurobehavioral symptoms and decreased cognitive performance.^6^ Despite lower posttraumatic stress scores, the symptom pattern elevated in combat crewmen suggests that occupational exposures may contribute to clinical presentation beyond what psychological factors alone explain. Differences in sustained neurotrauma may explain increased somatic, cognitive, vestibular, and headache symptoms. Despite no differences in the PSQI between groups, combat crewmen reported greater daytime sleepiness on the ESS. This may indicate reductions in functional alertness associated with persistent symptoms rather than sleep quality. These findings suggest a higher symptom burden among combat crewmen characterized by headache, fatigue, daytime sleepiness, cognitive, and vestibular complaints compared with special operators. The study is limited by its reliance on self-reported data and the fact that wave impacts are not uniformly experienced, as they vary by craft and environment. Further study is needed to detect neuroimaging, polysomnography, and neuropsychological correlates of combat crewmen exposures.

## Notes

Supplement 1. **eTable.** Measures, scores, cutoffs, and clinically relevant change

Supplement 2.
Data Sharing Statement
