# Supplementary material for: Clinical Characteristics of Combat Crewmen
Source: JAMA Netw Open. 2026 Mar 16;9(3):e262032. doi: 10.1001/jamanetworkopen.2026.2032 (PMC12993692; doi:10.1001/jamanetworkopen.2026.2032)
Supplement: Supplement 1. — eTable. Measures, scores, cutoffs, and clinically relevant change [file jamanetwopen-e262032-s001.pdf]

Supplemental Online Content

Powell JR, Lippa SM, McKenzie-Hartman TL, Sours Rhodes C, Pickett TC, Srikanchana R. Clinical characteristics of combat crewmen. *JAMA Netw Open*. 2026;9(3):e262032. doi:10.1001/jamanetworkopen.2026.2032

eTable. Measures, scores, cutoffs, and clinically relevant change

This supplemental material has been provided by the authors to give readers additional information about their work.

© 2026 Powell JR et al. *JAMA Network Open*.

eTable. Measures, scores, cutoffs, and clinically relevant change

Measure	Score Range	Interpretation of Score	Clinically Meaningful/Reliable Change
**Neurobehavioral Symptom Inventory (NSI)**	0–88	Higher score indicates greater symptom severity.	A change of 8 points is considered reliable.[1]
**PTSD Checklist - Military Version (PCL-M)**	17–85	Higher score indicates greater PTSD symptom severity.	A change of 5-10 points is considered reliable and a 10-20 point change is considered clinically meaningful.[2]
**Pittsburgh Sleep Quality Index (PSQI)**	0–21	Higher score indicates poorer sleep quality. A score >5 suggests significant sleep disturbance.[3]	A 3 point change is most frequently used as the minimal important change.[4]
**Epworth Sleepiness Scale (ESS)**	0–24	Higher score indicates greater daytime sleepiness. A score >11 suggests excessive daytime sleepiness.[5]	A 2-3 point decrease is considered the minimum clinically important change.[6]
**Patient Health Questionnaire-9 (PHQ-9)**	0–27	Higher score indicates greater depressive symptom severity. PHQ-9 scores of 5, 10, 15, and 20 represented mild, moderate, moderately severe, and severe depression, respectively.[7]	The minimum clinically important difference ranges from 5-9 points.[8]
**Generalized Anxiety Disorder-7 (GAD-7)**	0–21	Higher score indicates greater anxiety symptom severity.	A change of 4 points is considered clinically significant.[9]

© 2026 Powell JR et al. *JAMA Network Open*.

**Headache Impact Test (HIT-6)**	36–78	Higher score indicates greater adverse impact of headache on functioning.	The minimally important change was estimated to be -2.5 points [10]
**Alcohol Use Disorders Identification Test-Consumption (AUDIT-C)**	0–12	Higher score indicates a greater likelihood of hazardous drinking or active alcohol use disorder.	MCID is not typically used; Risk-level cutoffs are applied (e.g., ≥3 for women, ≥4 for men).

1. Belanger HG, Lange RT, Bailie J, et al. Interpreting change on the neurobehavioral symptom inventory and the PTSD checklist in military personnel. *Clin Neuropsychol.* 2016;30(7):1063-1073.
2. Monson CM, Gradus JL, Young-Xu Y, Schnurr PP, Price JL, Schumm JA. Change in posttraumatic stress disorder symptoms: do clinicians and patients agree? *Psychol Assess.* 2008;20(2):131-138.
3. Buysse DJ, Reynolds CF, 3rd, Monk TH, Berman SR, Kupfer DJ. The Pittsburgh Sleep Quality Index: a new instrument for psychiatric practice and research. *Psychiatry Res.* 1989;28(2):193-213.
4. Qin Z, Zhu Y, Shi DD, Chen R, Li S, Wu J. The gap between statistical and clinical significance: time to pay attention to clinical relevance in patient-reported outcome measures of insomnia. *BMC Med Res Methodol.* 2024;24(1):177.
5. Johns M, Hocking B. Daytime sleepiness and sleep habits of Australian workers. *Sleep.* 1997;20(10):844-849.
6. Patel S, Kon SSC, Nolan CM, et al. The Epworth Sleepiness Scale: Minimum Clinically Important Difference in Obstructive Sleep Apnea. *Am J Respir Crit Care Med.* 2018;197(7):961-963.
7. Kroenke K, Spitzer RL, Williams JB. The PHQ-9: validity of a brief depression severity measure. *J Gen Intern Med.* 2001;16(9):606-613.
8. Coley RY, Boggs JM, Beck A, Hartzler AL, Simon GE. Defining Success in Measurement-Based Care for Depression: A Comparison of Common Metrics. *Psychiatr Serv.* 2020;71(4):312-318.
9. Toussaint A, Husing P, Gumz A, et al. Sensitivity to change and minimal clinically important difference of the 7-item Generalized Anxiety Disorder Questionnaire (GAD-7). *J Affect Disord.* 2020;265:395-401.
10. Smelt AF, Assendelft WJ, Terwee CB, Ferrari MD, Blom JW. What is a clinically relevant change on the HIT-6 questionnaire? An estimation in a primary-care population of migraine patients. *Cephalalgia.* 2014;34(1):29-36.

© 2026 Powell JR et al. *JAMA Network Open*.
