# Supplementary material for: Clinical Characteristics of Combat Crewmen
Source: JAMA Netw Open. 2026 Mar 16;9(3):e262032. doi: 10.1001/jamanetworkopen.2026.2032 (PMC12993692; doi:10.1001/jamanetworkopen.2026.2032)
Supplement: Supplement 2. — Data Sharing Statement [file jamanetwopen-e262032-s002.pdf]

Data Sharing Statement

Data

Data available: No

Additional Information

Explanation for why data not available: Data from our sample of Naval Special Warfare service members cannot be made freely available due to operational security practices.
